# Acute Exposure to European Viper Bite in Children: Advocating for a Pediatric Approach

**DOI:** 10.3390/toxins13050330

**Published:** 2021-05-02

**Authors:** Marco Marano, Mara Pisani, Giorgio Zampini, Giuseppe Pontrelli, Marco Roversi

**Affiliations:** 1 Pediatric Intensive Care Unit, Pediatric Clinical Toxicology Center, Bambino Gesù Children’s Hospital, IRCCS, 00165 Rome, Italy; marco.marano@opbg.net (M.M.); giorgio.zampini@opbg.net (G.Z.); 2 Department of Emergency, Acceptance and General Pediatrics, Bambino Gesù Children’s Hospital, IRCCS, 00165 Rome, Italy; mara.pisani@opbg.net; 3 Clinical Trial Unit, Academic Department of Pediatrics, Bambino Gesù Children’s Hospital, IRCCS, 00165 Rome, Italy; giuseppe.pontrelli@opbg.net

**Keywords:** pediatrics, children, viper bite, envenoming, severity score, antivenom administration

## Abstract

Viper bite is an uncommon but serious cause of envenoming in Europe, especially in children. Our study aim is to better describe and analyze the clinical course and treatment of viper bite envenoming in a pediatric population. We retrospectively reviewed 24 cases of pediatric viper bites that were admitted to the Pediatric Emergency Department and the Pediatric Intensive Care Unit of the Bambino Gesù Children Hospital in Rome between 2000 and 2020. Epidemiological characteristics of the children, localization of the bite, clinical and laboratory findings, and treatment approaches were evaluated. The median age of the patients was 4.2 years, with male predominance. Most cases of viper bite occurred in the late summer. Most patients required admission to the ward for prolonged observation. The most common presenting signs were pain, local oedema, and swelling. Patients with a high severity score also had a significantly higher white blood cell count and an increase of INR, LDH, and CRP levels. No fatality was reported. Viper bite envenomation is a rare pediatric medical emergency in Italy but may sometimes be severe. A new pediatric severity score may be implemented in the screening of children with viper bites to favor a selective and prompt administration of antivenom.

## 1. Introduction

Snakebite envenoming is a rare yet potentially fatal event, reported to kill between 81,000 and 138,000 people and to cause long-lasting disabilities in another 400,000 people across the world [1]. Fortunately, snakebites are less common in Europe, occurring in every 0.22 to 1.43 people every 100,000 population per year in different countries [2]. According to the epidemiologic reports, fatalities following snakebite are rare events due to both the high-standard public health systems of these countries [3] and the lower lethality of the local snakes compared to their foreign counterparts, with the species *V.* *berus* the most involved, followed by the taxa *V. ammodytes* and *V. aspis*, and, less frequently, by the taxa *V. ursini*, *V. latastei*, and *V. seoanei* [4]. In Italy, *V. aspis* is the most common snake of the Viperidae family. About a quarter of snakebites occur without venom inoculation [5], as they are meant to scare the predator away, rather than killing the prey (‘dry’ bites). Given their lower weight-to-venom ratio, children are most exposed to the potentially lethal consequences of snakebites. Although the management of viper bite is mainly supportive, the cornerstone of the treatment is represented by immunotherapy, which should be implemented according to the severity scale (Table 1) developed by Audebert et al. [6], and later modified by Boels et al. [7]. However, few studies have reported data regarding children as a distinguished population and none has yet been published on an Italian cohort. The aim of this article is to report on the experience of a tertiary care pediatric hospital in Central Italy on 24 cases of viper snakebites in children and to suggest a grading severity score adapted to the pediatric age.

## 2. Results

All observed clinical and laboratory variables are reported in Table 2.

**Figure 1:**
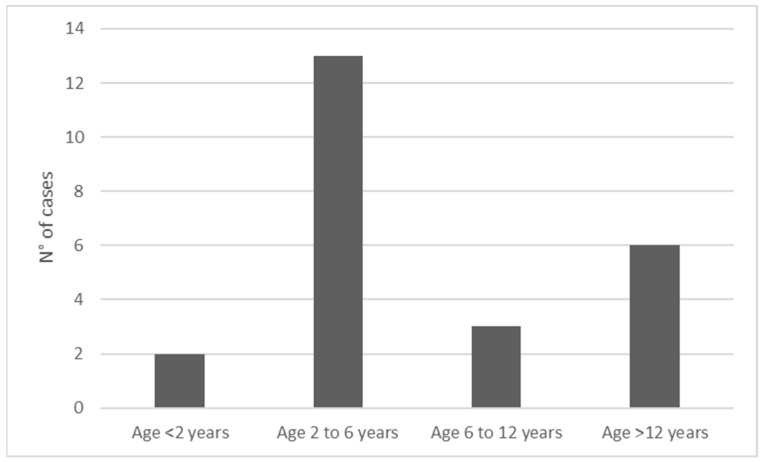
Age-groups and relative frequencies.

**Figure 2:**
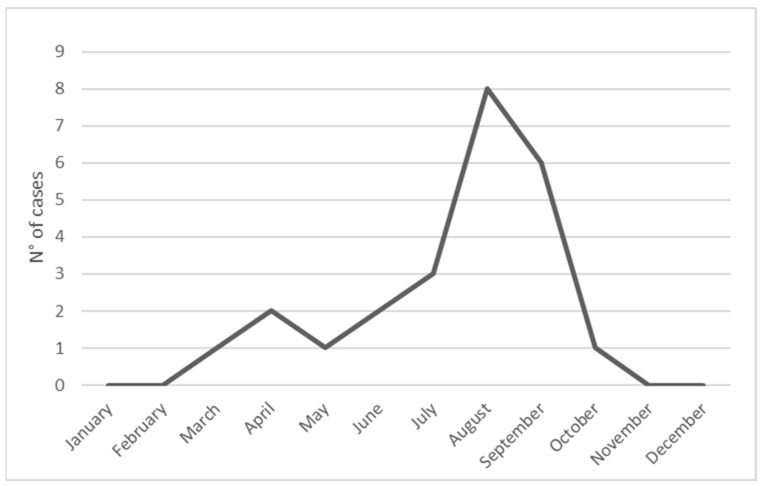
Monthly distribution of snakebites in children.

The median age of the patients was 4.2 years (range 1.5–16.2). Most patients were males (female to male ratio 1:1.6) and aged two to six years (54.2%) (Figure 1). Most cases of viper bite occurred in the late summer, especially in August and September (Figure 2), mainly in Central Italy. In three cases, the *V. aspis* species was identified (data not shown). All extremities were involved, with a slight predominance of the superior limbs (54.5%). Most patients required admission to the ward for prolonged observation (87.5%), with a median length of hospitalization of 3.5 days (range 0–14). No fatality was reported. The most common presenting signs were local edema and swelling (62.5%), along with recognizable fang marks. Perilesional and regional ecchymosis was less evident (33.3%). Two patients presented with ptosis and dysarthria or nystagmus. Only one patient lamented dyspnea. All patients complained of pain with different degrees of severity. Regarding the laboratory workup, leukocytosis with an abundance of neutrophils, elevated lactate dehydrogenase (LDH) levels and a slight increase in blood glucose and (international normalized ratio (INR) were found in the whole sample. No significant elevation of creatin phosphokinase (CPK) or activated plasma thromboplastin time (aPTT) was detected. Except for two patients with an important increase, inflammatory C-reactive protein (CRP) levels were low in all remaining cases. Of the patients who were admitted to the ward, 12 had a grading severity score (GSS) score higher than or equal to two (55%) and nine had a GSS score lower than two (45%). Antivenom administration was necessary in 12 cases (55%), with four of these patients requiring a second dose. No side effects were reported (data not shown), except in one case of serum sickness that presented with a mild rash of both legs a week after the first administration of the antivenom produced by Biomed. In four cases, low molecular weight heparin (LMWH) was also administered.

All observed variables were compared between the two groups of patients either with a GSS higher than or equal to two or a GSS lower than two, as depicted in Table 3.

When comparing the two groups, no significant difference was found in terms of median age, age group, gender, length of hospitalization, site of bite, or presenting signs and symptoms. A higher, but not significant (*p* = 0.087), prevalence of bites at the superior extremity was found in patients with a GSS score ≥ 2. A significant increase in white blood cells (*p* = 0.001), mainly neutrophils (*p* = 0.015), was found in this group as well as a significant increase of INR (*p* < 0.001), LDH (*p* = 0.035), and CRP (*p* = 0.034) levels. Not surprisingly, antivenom was significantly more administered in the same group.

When performing binary logistic regression focused on the results we previously obtained (data not shown) including white blood cells, neutrophil percentage, INR, LDH levels, site of bite, and all other variables with a *p*-value less than 0.2 as independent predictors of the outcome GSS ≥ 2, no significant finding was obtained. Given the great variability of CRP levels across the patients, we did not include it in this analysis.

Cut-off values of significant variables in the univariate analysis were calculated by the analysis of the relative ROC curves and are reported in Table 4. In particular, the following cutoffs proved significant: white blood cells count >11,000 cells/mm^3^, neutrophil percentage >65%, INR >1.15. The sensitivity, specificity, positive and negative predictive values for these cutoffs are reported in Table 5.

## 3. Discussion

Viper venom is a mixture of serine proteinases, metalloproteinases, and phospholipases A2 meant to harm, kill, and prepare the prey for digestion at the same time [8]. The cytotoxicity and myotoxicity of these enzymes is responsible for local pain, necrosis at the site of injection, and the regional edema and ecchymosis caused by disruption of blood vessel integrity [8]. Compared to the elapids, viper snake venom is less neurotoxic, while exhibiting more hemorrhagic capacity, due directly to the interference with the coagulation cascade and, indirectly, to the consumption of platelets and coagulation factors at the site of injection [9]. Although conflicting evidence proves variations in snake venom composition and lethality within the same species and/or for a given species with regard to age, sex, and milking season, an old study by Brown et al. reported an LD50 of all viper snakes ranging from 1.0 to 2.0 mg/kg, whether the injection was intravenous or subcutaneous, respectively [10]. Since the venom yield of such snakes is reported to not exceed 10 mg daily [11], the amount necessary to kill half of the adult males that came into contact with it (considering an average weight of 70 kg) would be that injected by 7 to 14 full bites. However, given their lower weight-to-venom ratio, children are more exposed to the potentially lethal consequences of snakebites. Among the factors associated with high-grade envenomation in children, Claudet et al. reported female gender, intense pain at onset, and being bitten in the afternoon [12], along with elevated glycemia and an upper-extremity location (confirmed by univariates analysis). Some studies have also suggested that developing countries experience higher mortality and morbidity associated with snakebites due to a lack of infrastructure, long referral times, and late administration of antivenom, in addition to the higher venom potency of the autochthonous snake species [3,13]. In a recent study comparing snakebites between adults and children, the latter were reported to have a significantly increased risk of edema, ecchymosis, sweating, and dyspnea from pulmonary edema (occurring in up to a third of all pediatric cases) compared to the former [14]. Other signs and symptoms of snake envenomation in children include fever, skin rash, diarrhea, vomiting, pain, and swelling of the affected limb, which may be so severe as to cause a compartment syndrome requiring fasciotomy (according to our experience, prompt administration of antivenom seems to reduce the risk of this complication). Anaphylactoid reaction, hypotension, and bronchospasm are also reported, but they may also occur after administration of the antivenom as an acute or delayed reaction (serum sickness). Pulmonary edema should be suspected when the swelling reaches the abdomen or thoracic wall or whenever dyspnea is appreciated [15]. Neurotoxic effects, with signs of involvement of the cranial nerves (ptosis and blurred vision) are well documented in the Mediterranean area [16,17,18]. In adults, acute myocardial infarction and renal hematoma have also been described [19,20], while no such pediatric cases have been reported in Europe, in accordance with our experience. Antivenoms are normally obtained from the sera of equins or ovins injected with the venom. These sera contained antibody fragments (with the Fab and without the Fc component of whole immunoglobulins) that showed preclinical efficacy against many species of viper snakes. However, clinical data on the results of antivenom administration is fragmented and often lacking [21].

The severity assessment of viper bites in the pediatric emergency department should be very attentive, especially in the first 24 h, given the potentially quick diffusion of the venom and its clinical effects. Antivenom administration is always recommended and should take place as soon as possible (under medical supervision) when the GSS score reaches grade 2 or more, in order to prevent progression of the illness. This score was developed by Boels et al. [5] by combining Audebert clinical grading with the laboratory values reported in another study conducted on adults [22]. Our study provides more insights on the clinical and laboratory characteristics of snake-bitten children. In these patients, we demonstrated that a higher level of white blood cells with prevalence of neutrophils and an increased INR are significantly correlated with the clinical severity of the envenomation. When comparing clinical and laboratory outcomes of children with high and low GSS, many *p*-values were very low, despite not reaching significance. This could mean that a sample with more individuals may produce significant results with the studied variables. The multivariate analysis did not significantly prioritize any predictor over the other, probably due to the collinearity of the factors analyzed.

**Figure 3:**
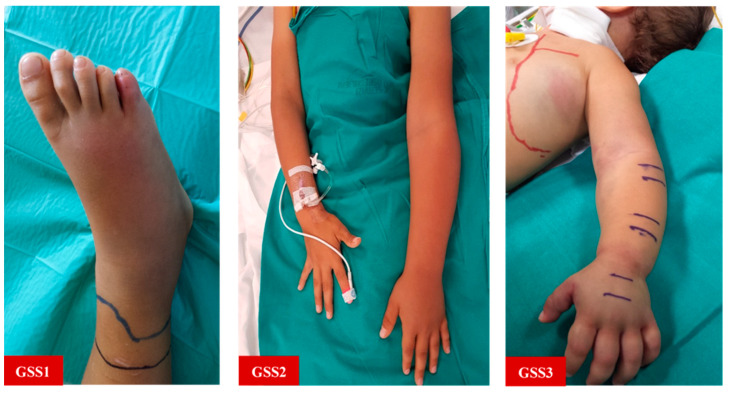
Clinical pictures. GSS1: Local edema around the site of bite; GSS2: Regional edema involving most of the bitten limb; GSS3: Extensive edema spreading into the trunk.

The cutoff values of significant variables discriminating for a GSS ≥ 2 or <2 were also calculated. Notably, we found a high negative predictive value (100%) for a neutrophil count <65%, meaning that no child with such a finding was assessed as clinically severe in our cohort. We embedded the significant cutoff values in a new pediatric grading severity score (pGSS), which also accounts for other specificities of pediatric pathology (Table 6 and Figure 3). In fact, we agree that children with a pGSS score higher than or equal to 2 should always be treated with administration of antivenom, addressing them to the intensive care unit whenever the edema spreads to the trunk or signs of hemodynamic instability appear (grade 3). The choice of the antivenom may be the object of controversy. At the time of writing, the EMA had not yet approved any of the antivenoms used in the study, given the lack of clinical trials. Given the large time span of the study, it should not surprise the reader that the use of obsolete yet effective sera such as the ‘Zagreb’ antivenom, which is not produced anymore. However, while polyvalent sera (that is, derived from multiple viper species) are effective antivenoms for the management of the main European viper species [21], other monovalent antivenoms may also be administered on the grounds of their cross-reactivity [23]. That being said, the ‘Viperfav’ (manufacturer: Sanofi-Pasteur MSD, France) antivenom, which is derived from *V. ammodytes*, *V. berus*, and *V. aspis* venom, seems to be the best choice for the treatment of viper bites in Italy, as in Europe, where these viper species reside. Of course, this choice should not delay administration of monovalent antivenom in children such as the ‘Biomed’ viper venom antitoxin (manufacturer: Biomed Sera and Vaccines, Poland), the ‘Bulbio’ snake venom antiserum (manufacturer: NCIPID Ltd., Bulgaria), the ‘Viekvin’ antivenom (manufacturer: Institute for Virology, Vaccines and Sera, “Torlak”, Serbia), and the ViperaTAb (manufacturer: Micro-Pharm Ltd., UK), when available for use.

The varying degree of pain experienced by the patients included in the study was not reported. However, pain is an important indicator of the quality of the bite. If the pain is severe, envenomation cannot be excluded and the patient should be monitored for at least 24 h, in order to check for symptom progression [24]. On the other hand, mild or no pain in the absence of other signs of envenomation is suggestive of a “dry bite”, with absent or minimal venom injection. In this case, a 6-h surveillance period in the emergency room may be proposed.

We also remind that pain should be closely monitored in children (and treated accordingly), as it may worsen in the case of a compartment syndrome. It is a common practice to administer antibiotics when suspecting a contaminated snakebite. Moreover, the same laboratory values that are indicative of infection also rise in the case of viper bites. Nonetheless, administration of antibiotics is not mandatory and should be decided according to the patient’s history, clinical examination, and laboratory workup together [5]. The use of corticosteroids is not indicated as it does not reduce the length of hospitalization, while predisposing the patient to secondary infection [5]. In the adult population, a tetanus vaccination booster is usually administered. In children, vaccine schedules should be examined, and serum dosage of tetanus antibodies performed in the case of uncertainty, before administering a vaccine boost. The administration of anticoagulants in viper bites is controversial. Some reports argue that LMWH might increase the risk of hematoma and functional impairment, especially when patients had received early antivenom treatment [5,25]. In a recent article [26], Boels et al. proved that a preventive administration of LMWH was significantly associated with a hospital stay of longer than 48 h. Nonetheless, in our experience, none of the 12 patients with a GSS ≥ 2 was discharged before 48 h from admission, with only four receiving LMWH. Therefore, we advise against the preventive use of LMWH, especially when an overt hemorrhage or bleeding disorder are evident, but we suggest its administration when direct evidence of thrombophlebitis is available or in cases of extensive edema, dehydration, decreased mobility, and/or prolonged decubitus, as in bedridden children admitted to the Pediatric Intensive Care Unit (PICU) on the grounds of an anticipated hospitalization longer than 48 h [27,28].

Our study has two major limits. First, we reported “only” 24 cases of snakebite in children, despite analyzing a period of many years. Snakebites, although quite frequent in adults, are still a rare event in children, as they constitute a fraction of an overall population that is increasingly leaving the countryside for the cities. Moreover, our report was monocentric and only multicentric studies conducted on a larger number of individuals may allow generalization of the observations we made.

Nonetheless, our study has an important strength: it is the first to have analyzed the largest cohort of snake-bitten children in Italy, thus offering a unique view on the characteristics of this rare yet dreading occurrence that any pediatrician, especially when far from cities and specialized facilities, may have to rapidly address.

## 4. Conclusions

Our study assessed the clinical and laboratory characteristics of viper bites in the largest Italian cohort of children treated in a specialized pediatric facility. It is also the first study to propose the implementation of a different grading severity score for snake-bitten children (pGSS) including the specific cutoff values of white blood cells, neutrophil percentage, and INR, and also proposes management indications, which could prove to be a ready-to-use tool in clinical practice in the case of snakebite for all emergency pediatricians. However, we strongly believe that our findings should be further validated through studies with a multicentric design involving larger cohorts of snake-bitten children.

## 5. Materials and Methods

We retrospectively reviewed 24 cases of snakebites in children admitted to the Pediatric Emergency Department and the Pediatric Intensive Care Unit of the Bambino Gesù Children Hospital in Rome between 2000 and 2020. The patients were evaluated according to the Grading Severity Score (GSS) of Audebert modified by Boels and colleagues [7]. For each patient, we reported the following clinical and laboratory variables (when available): age; age group (lower than two years; between two and six years; between six and 10 years; over 10 years); gender; days of hospitalization; anatomical site of bite (superior or inferior extremity); symptoms at onset including cutaneous (edema/swelling; ecchymosis; fang marks), respiratory, and neurologic signs (ptosis; dysarthria; nystagmus); hemoglobin level (g/dL); platelet count (cells/mm^3^); white blood cells (cells/mm^3^); neutrophils (%); eosinophils (%); blood glucose (mg/dL); LDH level (UI/L); CPK level (UI/L); fibrinogen level (mg/dL); D-dimers level (mcg/mL); INR; aPTT (seconds); CRP (mg/dL); administration of LMWH; administration/doses of intravenous antivenom. The sera administered were the antivenoms ‘Zagreb’ (manufacturer: Institute of Immunology Inc., Zagreb, Croatia), ‘Viekvin’ (manufacturer: Institute of Virology, Vaccines, and Sera “Torlak”, Belgrade, Serbia), and ‘Viper venom antitoxin’ (manufacturer: Biomed, Warsaw, Poland).All continuous data were expressed as means and standard deviations (if normally distributed) or as medians and ranges (if non-normally distributed). All categorical variables were expressed as proportions and percentages. The patients were divided into two groups, according to their GSS score: higher or equal to 2 and lower than 2. Subgroup analysis was performed with the Student t-test for normally distributed continuous variables, the Mann–Whitney U test for non-normally distributed continuous variables and the Chi-squared test for categorical variables. Multivariate analysis was performed via binary logistic regression, adopting the GSS score (higher than or equal to 2 and lower than 2, respectively) as the dependent variable and the significant results of the univariate analysis as the independent variables. A *p*-value less than 0.05 was considered statistically significant. Optimal cut-off values for a high or low GSS score were defined by receiver-operating characteristic (ROC) curve analysis by identifying the values with the highest Youden index and validating them on the same cohort with the Chi-squared test. Finally, we reported on the sensitivity, specificity, positive and negative predictive values of the significant cutoffs. The software IBM SPSS version 23.0 was used for statistical analysis. This retrospective study did not required approval by our Ethics Committee. Parents or legal guardians of participants of the study signed a consent form upon admission to the hospital for the anonymous use of their data for research purposes. Consent for publication of all pictures was obtained from the patients.

## Figures and Tables

**Table 1:** Audebert Classification modified by Boels et al.

Grade Name	Characteristics/Symptoms
0–No envenoming (“dry bite”)	Fang marks
	No oedema
	No local reaction
1–Minimal envenoming	Local oedema around the bite area
	No systemic symptoms
2–Moderate envenoming	Regional oedema involving a major part of limb
	Moderate systemic symptoms (slight hypotension, vomiting, diarrhoea)
	**2a** Regional oedema (most of the bitten limb) and/or haematoma, adenopathy
	**2b** Grade 2a + moderate general symptoms: mild hypotension, vomiting, diarrhoea, neurotoxic signs and/or biological criteria for severity:
	- Thrombocytes <150,000/L
	- Leukocytes >15,000/L
	- INR >1.5
	- Fibrinogen <2 g/L
3–Severe envenoming	Extensive oedema spreading into the trunk
	Severe systemic symptoms (prolonged hypotension, shock, bleeding)

**Table 2:** Demographic and clinical characteristics.

Parameters		Parameters	
Total population–no.	24	Hemoglobin (g/dL)–mean (SD)	12.1 (3.1)
Median age (range)–years	4.2 (1.5–16.2)	Platelet count (cells / mm^3^)–mean (SD)	340,900 (83,456)
Sex–no. (%)		White blood cells (cells / mm^3^)–mean (SD)	13,522 (8032)
Female	9 (37.56)	Neutrophils (% of WBCs)–mean (SD)	72 (18)
Male	15 (62.5)	Eosinophils (% of WBCs)–mean (SD)	0.95 (0.01)
Female to male ratio	1:1.6	Blood glucose (mg/dL)–mean (SD)	120 (67)
Admitted to ward–no. (%)	21 (87.5)	LDH (IU/l)–mean (SD)	618 (264)
Hospitalization (days)–median (range)	3.5 (0–14)	CPK (IU/l)–median (range)	351 (38–3722)
Site of bite		Fibrinogen (mg/dL)–mean (SD)	240 (85.5)
Superior extremity	12 (54.5)	D-dimers (μg/mL)–median (range)	0.7 (0.22–9.48)
Inferior extremity	10 (45.4)	INR–mean (SD)	1.16 (0.11)
Symptoms at onset–no. (%)		aPTT (seconds)–mean (SD)	30.3 (4.76)
Cutaneous signs	19 (79.2)	CRP (mg/dL)–median (range)	0.09 (0.05–14.4)
Oedema/swelling	15 (62.5)	GSS ≥ 2–no./total (%)	12/22 (55)
Ecchymosis	8 (33.3)	GSS < 2–no./total (%)	9/22 (45)
Fangs marks	13 (54.2)	LMWH administration–no./total (%)	4/19 (21)
Respiratory signs	1 (4.2)	Antivenom administration–no./total (%)	12/22 (55)
Neurologic signs	2 (8.3)	1 dose	8/12 (66.6)
Ptosis	2 (8.3)	2 doses	4/12 (33.3)
Dysarthria	1 (4.2)		
Nystagmus	1 (4.2)		

**Table 3:** Comparison of patients with high (≥2) or low (<2) GSS (admitted to ward).

Parameters	GSS < 2	GSS ≥ 2	*p*-Value
Total population–no.	9 (42.9%)	12 (57.1%)	
Median age (range)–years	6.41 (3.2–8.81)	3.61 (1.47–13.41)	0.382
Age distribution–no. (%) *			
<2 years	0/9 (0)	2/12 (16.6)	0.486
2 to 6 years	5/9 (55.5)	6/12 (50)	1.000
6 to 12 years	2/9 (22.2)	1/12 (0.08)	0.553
>12 years	2/9 (22.2)	3/12 (25)	1.000
Sex–no. (%) *			
Males	4/9 (44.4)	10/12 (83.3)	0.331
Hospitalization (days)–mean (SD)	5.33 (3.06)	6.5 (3.75)	
Site of bite			
Superior extremity	3/9 (33)	9/12 (75)	0.087
Symptoms at onset–no. (%) *			
Cutaneous signs	7/9 (77.7)	12/12 (100)	0.171
Respiratory signs	0/9 (0)	1/12 (0.08)	1.000
Neurologic signs	0/9 (0)	2/12 (16.6)	0.486
Blood parameters			
Hemoglobin–mean (SD)	12.2 (0.9)	12.1 (4.1)	0.898
Platelet count–mean (SD)	333,780 (84,602)	346,250 (85,938)	0.744
White blood cells–mean (SD)	8231 (3140)	18,618 (6938)	0.001
Neutrophils %–mean (SD)	58 (2.1)	82 (9)	0.015
Eosinophils %–mean (SD)	1.6 (1.1)	0.5 (0.5)	0.031
Blood glucose–mean (SD)	94 (13)	138 (83)	0.161
LDH–mean (SD)	504 (96)	694 (315)	0.035
CPK–median (range)	134 (38–213)	172 (82–3722)	0.270
Fibrinogen–mean (SD)	280.5 (62)	244 (84)	0.285
D-dimers–median (range)	0.56 (0.22–1.56)	0.74 (0.28–9.48)	0.066
INR–mean (SD)	1.07 (0.06)	1.24 (0.08)	<0.001
aPTT–mean (SD)	30.2 (2.4)	30.4 (6.1)	0.451
CRP–median (range)	0.08 (0.05–0.75)	0.18 (0.05–14.4)	0.034
LMWH administration–no./total (%)	1/9 (11.1%)	3/9 (33.3%)	0.288
Antivenom administration–no./total (%)	0/9 (0%)	12/12 (100%)	<0.001

* Percentages were calculated excluding missing data.

**Table 4:** Sensitivity, specificity, positive and negative predictive values of significant cutoffs for a GSS ≥ 2.

Parameters	Sensitivity	Specificity	PPV	NPV
GB > 11,000 cell/mm^3^	92%	78%	88%	85%
*n* > 65%	100%	67%	50%	100%
INR > 1.15	92%	78%	89%	92%

**Table 5:** Cut-off values of significant variables (for a GSS ≥ 2).

Cut-Offs	*p*-Value
GB > 11,000 cell/mm^3^	0.002
*n* > 65%	0.004
INR > 1.15	0.003
LDH > 530 UI/L	0.065
Blood glucose >100 mg/dL	0.065
Bite at the superior extremity	0.084

**Table 6:** Audebert–Boels classification adapted to children by Marano et al.

Grade Name	Characteristics/Symptoms	Suggested Interventions
0–No envenoming (“dry bite”)	Fang marks No edema No local reaction	➔ 6-h surveillance in the emergency room
1–Minimal envenoming	Local edema around the bite area No systemic symptoms	➔ Clinical observation up to evident reduction of edema ➔ Supportive care, including hydration and pain relief
2–Moderate envenoming	**Grade 2a** One or both of the following: - Regional edema with progression to most of the limb - Hematoma or adenopathy **Grade 2b** Grade 2a + moderate general symptoms (mild hypotension, vomiting, diarrhea, neurotoxic signs) and/or biological criteria for severity: - Leucocytes > 11,000/L - Neutrophils > 65% - INR > 1.15	➔ Clinical observation up to the evident reduction of edema (evaluate district perfusion and saturation) ➔ Supportive care, including hydration and pain relief ➔ Doppler-ultrasound of affected limb’s blood vessels ➔ Administration of antivenom ➔ Evaluate antibiotic therapy * ➔ Administer LMWH **
3–Severe envenoming	One or both of the following: - Edema spreading to the trunk - Signs of hemodynamic instability (prolonged hypotension, shock, bleeding)	➔ Same interventions as in Grade 2 ➔ Admission to PICU

* Only if clinical or laboratory signs of bacterial contamination are evident, ** Only if direct evidence of thrombophlebitis is available or in cases of extensive edema; dehydration; decreased mobility; prolonged decubitus; admission to PICU; anticipated hospitalization longer than 48 h. Do not administer in the case of overt hemorrhage or a bleeding disorder.

## Data Availability

The data presented in this study are available on request from the first author [MM].
